# Comparison of 2002 AECG and 2016 ACR/EULAR classification criteria and added value of salivary gland ultrasonography in a patient cohort with suspected primary Sjögren’s syndrome

**DOI:** 10.1186/s13075-017-1475-x

**Published:** 2017-12-06

**Authors:** Maëlle Le Goff, Divi Cornec, Sandrine Jousse-Joulin, Dewi Guellec, Sebastian Costa, Thierry Marhadour, Rozenn Le Berre, Steeve Genestet, Béatrice Cochener, Sylvie Boisrame-Gastrin, Yves Renaudineau, Jacques-Olivier Pers, Alain Saraux, Valérie Devauchelle-Pensec

**Affiliations:** 1 Centre National de Référence CERAINO, Service de Rhumatologie, Hôpital de la Cavale Blanche, BP 824, F 29609 Brest cedex, France; 2 INSERM UMR1227, Lymphocytes B et Autoimmunité, Université de Bretagne Occidentale, Brest, France; 3 Anatomie et Pathologie, CHRU Brest, Brest, France; 4 Médecine Interne, CHRU Brest, Brest, France; 5 Explorations Fonctionnelles Neurologiques, CHRU Brest, Brest, France; 6 Ophtalmologie, CHRU Brest, Brest, France; 7 Odontologie, CHRU Brest, Brest, France; 8 Laboratoire d’Immunologie et Immunothérapie, CHRU Brest, Brest, France

**Keywords:** Sjögren’s syndrome, Primary, Classification criteria, Diagnosis, Ultrasonography

## Abstract

**Background:**

The objective was to evaluate concordance between 2002 American-European Consensus Group (AECG) and 2016 American College of Rheumatology (ACR)/European League Against Rheumatism (EULAR) classification criteria for primary Sjögren’s syndrome (pSS) and to assess how salivary gland ultrasonography (SGUS) might improve the classification of patients.

**Methods:**

Patients with suspected pSS underwent a standardised evaluation, including SGUS, at inclusion into the single-centre Brittany DIApSS cohort. Agreement between the two criteria sets was assessed using Cohen’s κ coefficient. Characteristics of discordantly categorised patients were detailed.

**Results:**

We prospectively included 290 patients between 2006 and 2016, among whom 125 (43%) met ACR/EULAR criteria and 114 (39%) also met AECG criteria; thus, 11 (4%) patients fulfilled only ACR/EULAR, no patients AECG only, and 165 (57%) patients neither criteria set. Concordance was excellent (κ = 0.92). Compared to patients fulfilling both criteria sets, the 11 patients fulfilling only ACR/EULAR criteria had similar age and symptom duration but lower frequencies of xerophthalmia and xerostomia (*p* < 0.01 for each) and salivary gland dysfunction (*p* < 0.01); most had systemic involvement (91%), including three (27%) with no sicca symptoms; 91% had abnormal salivary gland biopsy and 46% anti-Sjögren's-syndrome-related antigen A (anti-SSA); 64% were diagnosed with pSS by the physician. SGUS was abnormal in 12% of the 165 patients fulfilling no criteria set. Including SGUS among the ACR/EULAR criteria increased sensitivity from 87.4% to 91.1% when physician diagnosis was the reference standard.

**Conclusions:**

Agreement between AECG and ACR/EULAR criteria sets is excellent. ACR/EULAR criteria are slightly more sensitive and classified some patients without sicca symptoms as having pSS. Including SGUS in the ACR/EULAR criteria may further improve their sensitivity.

## Background

Primary Sjögren’s syndrome (pSS) is a chronic systemic auto-immune inflammatory disease characterised by secretory gland dysfunction leading to oral and/or ocular dryness in most patients. Furthermore, 30–50% of patients with pSS exhibit a broad spectrum of systemic manifestations [1]. The prevalence in the general population is 0.02–0.1%, and middle-aged women are predominantly affected [2–4]. Although mortality is not higher in patients with pSS than in the general population [5], the cardinal symptoms of ocular and oral dryness, fatigue, and diffuse pain severely diminish quality of life [6]. Despite recent insights into the pathophysiology of pSS [7], no treatment has been demonstrated to improve the course of the disease [8].

Over the last few decades, many classification systems have been developed to define pSS and assist in research and clinical practice. The set of subjective and objective criteria issued by the American-European Consensus Group (AECG) in 2002 has been the main classification system used in clinical studies during the last decade [9]. In 2012, the Sjögren’s International Collaborative Clinical Alliance (SICCA) [10] issued new classification criteria, which were first endorsed by the American College of Rheumatology (ACR) [11]. Several studies then identified difficulties raised by the co-existence of the two criteria sets [12–14]. New consensual classification criteria for pSS combining features of the earlier ACR and AECG criteria sets were therefore developed and validated jointly by ACR and EULAR committees [15, 16]. This ACR/EULAR criteria set excludes the most common differential diagnoses. It also differs substantially from the earlier AECG criteria (Table 1) in that it considers systemic manifestations (defined as a EULAR Sjögren’s Syndrome Disease Activity Index (ESSDAI) ≥ 1) [17, 18], and sicca symptoms, as entry criteria. A weighted scoring system is then applied, with 3 points each for positive salivary gland biopsy (SGB) [19, 20] and positive anti-SSA antibodies and 1 point each for unstimulated whole salivary flow (UWSF) ≤ 0.1 mL/min [21], Schirmer’s test result ≤ 5 mm/5 min and Ocular Staining Score (OSS) ≥ 5 [22] or van Bijsterveld (VB) score ≥ 4. A weighted score ≥ 4 classifies the patient as having pSS.

**Table 1:** AECG versus ACR/EULAR criteria

	2002 AECG criteria	2016 ACR/EULAR criteria	
			Weight
Items	1. Ocular dryness symptoms 2. Oral dryness symptoms 3. Ocular signs: Schirmer’s test ≤ 5 mm/5 min or van Bijsterveld score ≥ 4 4. Focus score ≥ 1 foci/4 mm^2^ on minor salivary gland biopsy 5. Salivary gland involvement: unstimulated whole salivary flow ≤ 0.1 mL/min 6. Positive anti-SSA or SSB antibodies	1. Labial salivary gland with focal lymphocytic sialadenitis and focus score of ≥ 1 foci/4 mm^2^	3
		2. Anti-SSA/Ro-positive	3
		3. Ocular Staining Score ≥ 5 (or van Bijsterveld score ≥ 4) in at least one eye	1
		4. Schirmer’s test ≤ 5 mm/5 min in at least one eye	1
		5. Unstimulated whole saliva flow rate ≤ 0.1 mL/min	1
Rules for classification	− Absence of exclusion criteria^a^ − Presence of any 4 of the 6 items with at least item 4 or 6, or − presence of any 3 of the 4 objective items (3, 4, 5, and 6)	Applies to any individual	
		− who meets the inclusion criteria^b^ with at least one symptom of ocular or oral dryness or ESSDAI ≥ 1 − does not have any of the conditions listed as exclusion criteria^c^ − and has a score of ≥ 4 when the weights from the 5 criteria items are summed	

*AECG* American-European Consensus Group, *ACR* American College of Rheumatology, *SS* Sjögren’s syndrome, *ESSDAI* EULAR SS Disease Activity Index, *anti-SSA* anti-Sjögren's-syndrome-related antigen A

^a^Exclusion criteria in the AECG system: past head and neck radiation treatment, hepatitis C infection, acquired immunodeficiency syndrome (AIDS), pre-existing lymphoma, sarcoidosis, graft-versus-host disease, use of anticholinergic drugs (within a time shorter than fourfold the half-life of the drug)

^b^These criteria are applicable to any patient with at least one symptom of ocular or oral dryness or in whom there is a suspicion of Sjögren’s syndrome (SS) based on the ESSDAI (at least one domain with a positive item).

^c^Exclusion criteria for ACR/EULAR criteria include a prior diagnosis of any of the following conditions, which would exclude diagnosis of SS and participation in SS studies or therapeutic trials because of overlapping clinical features or interference with criteria tests: history of head and neck radiation treatment, active hepatitis C infection (with confirmation by PCR), AIDS, sarcoidosis, amyloidosis, graft-versus-host disease, and IgG4-related disease

Several recent studies assessed major salivary gland ultrasonography (SGUS) as a tool for diagnosing pSS [23–28]. Including SGUS in the AECG and ACR criteria sets may improve performance [23, 24]. However, SGUS is not among the ACR/EULAR criteria, because the procedure was not performed in the patients included in the cohorts used to develop and validate the criteria set.

The concordance and differences in the results of the AECG and ACR/EULAR criteria sets in independent patient populations must be evaluated to aid in interpreting comparisons of future clinical studies based on the new criteria set to previously published studies. Here, our objectives were to assess agreement between the two criteria sets, to identify sources of disagreement, and to analyse SGUS findings according to patient classification.

## Methods

### Inclusion and exclusion criteria

We conducted a cross-sectional study in the single-centre Brittany cohort of patients with suspected pSS (DIApSS cohort). Patients were included prospectively between January 2006 and September 2016 at the Brest University Hospital, Brest, France. As previously described [23, 29], patients were included if they had subjective ocular and/or oral dryness, major salivary gland swelling, extra-glandular manifestations consistent with pSS, or suggestive antibodies or other laboratory abnormalities. Patients were referred to our multidisciplinary clinics by their family physician, rheumatologist, internist, oral health specialist, or ophthalmologist. We excluded patients with a diagnosis of another connective tissue disease. All participants gave written informed consent, and the study was approved by the Brest University Hospital institutional review board.

### Standardised evaluation

All patients underwent a comprehensive standardised clinical evaluation conducted by an experienced rheumatologist, an oral health specialist [30], and an ophthalmologist. UWSF ≤ 0.1 mL/minute [21], Schirmer’s test result ≤ 5 mm/5 minutes, and VB score ≥ 4 in at least one eye [31] were considered abnormal. All patients underwent standard laboratory tests, immunological tests (anti-nuclear antibodies, anti-SSA, anti-SSB, and rheumatoid factors, as previously described [32, 33]), and minor labial SGB. The rheumatologist determined the most probable diagnosis and assessed the clinical probability of pSS from 1 (definitely not pSS) to 4 (definitely pSS). All doubtful cases (two (probably not pSS) and 3 (probably pSS)) were reviewed by a panel of three experts (VD-P, AS, and SJJ) to reach a consensus. B-mode SGUS was performed by a single experienced operator (SJJ), who was blinded to the diagnosis and scored the echo-structure from 0 to 4 for each of the four major salivary glands (two parotid and two submandibular glands). The highest grade was recorded and was considered abnormal if ≥ 2, as previously described [34].

### Statistical analysis

Statistical tests were performed using the Statistical Package for the Social Sciences (SPSS 20.0; SPSS Inc., Chicago, IL, USA). Quantitative variables were described as mean ± standard deviation and qualitative variables as number (percentage). Classification criteria were applied to each patient as described in Table 1 (only the VB score was used to apply ACR/EULAR criteria because the OSS was unavailable for most patients). Taking the physician’s diagnosis as the reference standard for defining cases may lead to overestimation of the diagnostic performance of a classification system that has previously been used in everyday practice, with a risk of circular reasoning. Consequently, in our primary analysis, we compared patient groups defined by the two criteria sets. Agreement between classification criteria sets, and between classification criteria sets and physician diagnosis, was evaluated using Cohen’s kappa coefficient (κ). To compare patient groups, we used the Mann-Whitney test, Fisher’s exact test, or the chi-square test as appropriate. The characteristics of discordantly classified patients were detailed.

## Results

### Cohort characteristics

Between January 2006 and September 2016, 324 patients were included prospectively in the DIApSS cohort. Among them, 34 were excluded from the present study because they were diagnosed with, and met classification criteria for, another connective tissue disease (mainly rheumatoid arthritis and systemic lupus erythematosus). Thus, 290 patients were analysed in this study. Mean age was 55.8 ± 13.4 years, 92% (*n* = 267) were female, mean symptom duration was 6.4 ± 7.1 years, and 47% (n = 135) received a physician diagnosis of pSS.

### Comparison of ACR/EULAR and AECG criteria

Table 2 compares patients meeting 2016 ACR/EULAR criteria and/or 2002 AECG criteria. More patients fulfilled ACR/EULAR criteria (*n* = 125, 43.1%) than AECG criteria (*n* = 114, 39.3%). All 114 patients meeting AECG criteria also met ACR/EULAR criteria (positive concordant group), whereas 11 (3.8%) patients met only ACR/EULAR criteria (discordant group). Finally, 165 (56.9%) patients met neither criteria set (negative concordant group). Agreement between the criteria sets was excellent (κ = 0.92).

**Table 2:** Comparison of patients meeting 2016 ACR/EULAR criteria and/or 2002 AECG criteria

	Patients fulfilling both sets of criteria *n* = 114	Patients fulfilling ACR/EULAR set of criteria only *n* = 11	Patients fulfilling neither set of criteria *n* = 165	*p* value*
Age (years, mean ± SD)	56.6 ± 13.7	53.6 ± 16.2	55.0 ± 12.6	0.56
Symptom duration (years, mean ± SD)	6.6 ± 7.1	5.5 ± 6.7	5.6 ± 6.2	0.46
Female, *n* (%)	106 (93)	11 (100)	150 (91)	1
Xerophthalmia^a^, *n* (%)	110 (97)	2 (18)	141 (86)	< 0.01
Xerostomia^a^, *n* (%)	111 (97)	6 (55)	144 (87)	< 0.01
ESSDAI (mean ± SD)	4.8 ± 5.5	4.6 ± 3.2	3. 8 ± 4.8	0.58
ESSDAI ≥ 1, *n* (%)	98 (86)	10 (91)	115 (70)	1.00
Only sicca, *n* (%)	16 (14)	1 (9)	52 (32)	1.00
Only ESSDAI, *n* (%)	1 (1)	3 (27)	6 (4)	< 0.01
Schirmer’s test ≤ 5 mm/5 min, *n* (%)	70/110 (64)	4 (36)	47/154 (31)	0.18
VB ≥ 4, *n* (%)	24/80 (30)	2/4 (50)	12/85 (14)	0.58
UWSF ≤ 0.1 mL/min, *n* (%)	73/103 (71)	2 (18)	56/152 (37)	< 0.01
Abnormal SGB, *n* (%)	93/112 (83)	10 (91)	22/159 (14)	0.69
Anti-SSA and/or SSB positivity, *n* (%)	77 (68)	5 (46)	6 (4)	0.14
Anti-SSA, *n* (%)	75 (66)	5 (46)	5 (3)	0.18
ANA ≥ 1:320, *n* (%)	83 (73)	8 (73)	55 (33)	1.00
RF positivity, *n* (%)	51/112 (46)	4 (36)	18/165 (11)	0.75
IgG (g/L, mean ± SD)	14.5 ± 7.9	14. 7 ± 5.1	10. 5 ± 3.5	0.49
SGUS score ≥ 2, *n* (%)	61/105 (58)	4/9 (44)	17/141 (12)	0.50
Physician diagnosis of pSS, *n* (%)	111 (97)	7 (64)	17 (10)	< 0.01

*ACR* American College of Rheumatology, *AECG* American-European Consensus Group, *ESSDAI* EULAR SS Disease Activity Index, *Anti-SSA* anti-Sjögren's-syndrome-related antigen A, *VB* van Bijsterveld score, *UWSF* unstimulated whole salivary flow, *SGB* salivary gland biopsy, *ANA* antinuclear antibodies, *RF* rheumatoid factor, *SGUS* salivary gland ultrasonography, pSS, primary Sjögren’s syndrome

*Comparison of patients fulfilling both criteria sets and of patients fulfilling only the ACR/EULAR criteria set

^a^Subjective complaints by the patients

Compared to the 114 patients fulfilling both criteria sets, the 11 discordant patients fulfilling ACR/EULAR but not AECG criteria had similar mean age (53.6 ± 16.2 versus 56.6 ± 13.7 years, *p* = 0.56) and mean symptom duration (5.5 ± 6.7 versus 6.6 ± 7.1 years, *p* = 0.46). The discordant group had lower prevalence of sicca symptoms (ocular dryness, 18.2% versus 96.5%, *p* < 0.01; and oral dryness, 54.5% versus 97.4%, *p* < 0.01) and salivary gland dysfunction (UWSF ≤0.1 mL/min: 18% versus 70.9%, *p* < 0.01). Of the 11 discordant patients, 10 (90.9%) had systemic involvement (ESSDAI ≥1); mean ESSDAI was similar in the discordant and positive concordant groups (4.6 ± 3.2 versus 4.8 ± 5.5, *p* = 0.58). Compared to the positive concordant group, a larger proportion of patients in the discordant group had ESSDAI ≥ 1 but no sicca symptoms (27.3% versus 0.9%, *p* < 0.01). In the discordant group, 10/11 (90.9%) patients had a positive SGB and 5/11 (45.4%) had anti-SSA and/or anti-SSB antibodies. In the overall cohort, two patients had anti-SSB but not anti-SSA antibodies, and therefore met the serological criterion in the AECG set but not the ACR/EULAR set. These two patients had typical features of pSS and fulfilled both AECG and ACR/EULAR criteria based on abnormal SGB and UWSF findings.

In patients meeting ACR/EULAR criteria, the main reasons for not also meeting AECG criteria were absence of sicca symptoms, presence of either xerophthalmia or xerostomia but not both, and presence of only two other criteria including anti-SSA or positive SGB. Of note, the VB score was available for only 4/11 discordant patients; among the remaining 7 patients, 4 had a negative Schirmer’s test: these 4 patients may have also fulfilled AECG criteria had a VB score been obtained and had it been positive (which was the case in only 30% of patients fulfilling AECG criteria, Table 2).

The physician diagnosed pSS in 7/11 (63.6%) discordant patients compared to 111/114 (97.4%) positive concordant patients (*p* < 0.01). When the physician’s diagnosis was used as the reference standard, the ACR/EULAR criteria had 87.4% sensitivity and 95.4% specificity for pSS, compared to 82.2% and 98.1%, respectively, for the AECG criteria. Concordance with the physician diagnosis was similar for the AECG and ACR/EULAR criteria (κ = 0.81 and κ = 0.83, respectively).

### Detailed features of patients fulfilling only ACR/EULAR criteria (*n* = 11)

Table 3 details the features of the 11 patients fulfilling only ACR/EULAR criteria. All were female. Among them, 10 had systemic activity (ESSDAI ≥ 1): 7 had inflammatory arthralgia, 2 cytopenia, 1 parotidomegaly, 1 lymphadenopathy, 1 peripheral axonal neuropathy, and 5 positive items in the biological ESSDAI domain (3 with moderate and 2 with low activity, and all 5 with involvement of other domains). Five patients had other organ-specific auto-immune diseases such as thyroiditis and hepatitis. In the four patients who did not receive a physician diagnosis of pSS, the physician felt that the most likely diagnosis was idiopathic sicca syndrome (*n* = 3) or undifferentiated inflammatory arthritis (*n* = 1).

**Table 3:** Description of the 11 patients fulfilling only ACR/EULAR criteria

Pt.	Age	Dura-tion^a^	Eye^b^	Mouth^c^	ESSDAI	ESSDAI domains^d^	Other AID	Low UWSF	Schirmer	VB ≥ 4	SGB	ANA	SSA	SSB	RF	IgG	SGUS	Diag^e^
1	51	1	0	1	4	A, B	0	1	0		1	0	0	0	1	23.7	1	1
2	38	2	0	1	2	A	0	0	1		1	320	0	0	1	11.5	0	0
3	80	12	0	1	0	/	Thyroiditis	1	0		1	320	0	0	0	12.1	0	1
4	54	1	0	0	5	A, G, B	0	0	0		1	1 280	1	0	0	17.5	1	1
5	19	6	0	0	10	PNS	0	0	0		1	320	1	0	1	12.5	1	1
6	61	5	1	0	2	A	Thyroiditis	0	0	1	1	0	0	0	1	9.5	1	0
7	64	23	1	0	6	A, H, B	AI hepatitis	0	0	0	1	320	1	0	0	15.8	/	1
8	57	4	0	1	10	A, H, B	AI hepatitis	0	1	1	0	1280	1	1	1	20.8	/	1
9	67	2	0	1	2	A	0	0	1	0	1	320	0	0	0	8	0	0
10	42	1	0	0	5	A, B	0	0	0		1	320	1	0	0	19	0	1
11	56	3	0	1	4	L	Thyroiditis	0	1		1	160	0	0	0	9.7	0	0

*Pt* patient, *ESSDAI* EULAR pSS Disease Activity Index, *AID* autoimmune disease, *UWSF* unstimulated whole salivary flow, *VB* van Bijsterveld score, *SGB* salivary gland biopsy, *ANA* anti-nuclear antibodies, SSA Anti-Sjögren's-syndrome-related antigen A *RF* rheumatoid factors, *SGUS* salivary gland ultrasonography

^a^Symptom duration in years

^b^Subjective ocular dryness

^c^Subjective oral dryness

^d^ *A* articular, *P* pulmonary, *PNS* peripheral nervous system, *R* renal, *G* glandular, *C* cutaneous, *L* lymphadenopathy, *H* hematological, *B* biological

^e^Diagnosis of primary Sjögren’s syndrome by the physician

### Detailed features of patients who had physician-diagnosed pSS but met neither of the criteria sets

Table 4 details the features of the 17 patients (16 females) who met neither of the criteria sets but received a diagnosis of pSS from the physician. All but one had sicca symptoms, 10 had recent-onset disease (defined as symptom duration ≤ 5 years), 12 had systemic involvement with no other explanation than pSS, 9 had an abnormal SGB, and 4 had anti-SSA/SSB antibodies. The main reason for not meeting criteria was absence of objective signs of ocular or oral dryness (only one patient had a positive Schirmer’s test and another an abnormal VB score). Four patients had a negative SGB and no anti-SSA; all four had sicca symptoms, typical systemic involvement (with no differential diagnosis), and a biological sign not included in the criteria set (high serum IgG levels, rheumatoid factors, or high-titre anti-nuclear antibodies).

**Table 4:** Description of the 17 patients who received a physician diagnosis of pSS but fulfilled neither classification criteria set

Pt.	Age	Duration^a^	Eye^b^	Mouth^c^	ESSDAI	ESSDAI domains^d^	Other AID	Low UWSF	Schirmer	VB ≥ 4	SGB	ANA	SSA/SSB	RF	IgG	SGUS
1	52	4	1	1	0	/	0	0	0	/	1	640	0	0	11.6	1
2	25	0	0	0	6	H	0	0	0	0	0	160	1	1	11.6	0
3	63	1	1	1	0	/	0	/	0	0	1	1 280	0	0	6,9	1
4	69	3	1	1	10	PNS, R	0	0	0	/	1	0	0	0	9,8	0
5	48	6	1	1	19	A, CNS, B	Thyroiditis	0	0	/	0	0	0	0	22.0	0
6	39	3	1	0	2	A	0	0	0	/	1	1 280	0	0	11.9	1
7	47	24	1	1	4	H, B	0	/	1	/	0	0	0	1	31.4	0
8	60	20	1	1	8	A, PNS, B	0	0	0	/	0	1 280	1	0	18.9	1
9	50	13	1	1	14	A, PNS, G	0	0	0	0	1	160	0	0	11.4	0
10	50	1	1	1	4	H	0	0	0	/	0	1 280	0	0	10.7	0
11	55	7	1	1	0	/	0	0	0	0	1	160	0	0	8.8	0
12	61	5	1	1	0	/	0	0	0	0	1	0	0	0	11.2	0
13	49	2	1	1	0	/	0	0	0	0	1	160	0	0	10.1	1
14	51	4	1	0	2	G	0	0	0	0	0	0	1	1	6.7	1
15	63	1	1	1	8	A, C	0	0	0	0	1	160	0	0	11.0	0
16	65	28	1	1	1	B	0	0	0	1	0	320	0	0	7.7	0
17	51	1	1	1	12	R, B	0	0	0	0	0	320	1	0	15.9	1

*pSS* primary Sjögren’s syndrome *Pt* patient, *ESSDAI* EULAR pSS Disease Activity Index, *AID* autoimmune disease, *UWSF* unstimulated whole salivary flow, *VB* van Bijsterveld score, *SGB* salivary gland biopsy, *ANA* anti-nuclear antibodies, *RF* rheumatoid factors, *SGUS* salivary gland ultrasonography, SSA Anti-Sjögren's-syndrome-related antigen A

^a^Symptom duration in years

^b^Subjective ocular dryness

^c^Subjective oral dryness

^d^ *A* articular, *P* pulmonary, *PNS* peripheral nervous system, *R* renal, *G* glandular, *C* cutaneous, *L* lymphadenopathy, *H* hematological, *B* biological

### Impact of salivary-gland ultrasonography (SGUS) on classification

Among the 290 patients in the cohort, 255 underwent SGUS, which was abnormal in 82 patients (31.2%). The proportion of patients with abnormal SGUS was 44.4% in the discordant group and 58.1% in the positive concordant group (*p* = 0.50). Among the 17 patients who met neither criteria set but received a physician diagnosis of pSS, 7 (41%) had SGUS abnormalities. These seven patients had either anti-SSA antibodies or abnormal SGB but did not fulfil the criteria set because of normal findings in Schirmer’s test, the VB score, and UWSF. This suggests that including SGUS in the ACR/EULAR criteria as an alternative procedure for objectively assessing exocrine gland involvement may further improve sensitivity. Only 8% of patients who did not receive a physician diagnosis of pSS had an abnormal SGUS, confirming the good specificity of the procedure. We tested the possibility of including SGUS among ACR/EULAR criteria, arbitrarily giving SGUS the same weight as UWSF, Schirmer’s test, and the VB score (1 point if positive) and using the same cutoff (≥ 4) to classify a patient as having pSS. Using a physician diagnosis of pSS as the reference standard, including SGUS inclusion among ACR/EULAR criteria slightly increased their sensitivity from 87.4% to 91.1% (absolute increase 3.7%), while the specificity remained over 90% (95.4% without and 93.8% with SGUS). Importantly, no patient fulfilled these modified criteria without positive SGB or anti-SSA.

## Discussion

In a prospective cohort of consecutive patients from everyday clinical practice, with sicca symptoms or systemic involvement suggesting pSS, agreement between AECG and ACR/EULAR criteria was excellent (κ = 0.92). Thus, these two criteria sets would select similar patient populations for future trials and clinical studies. This excellent agreement is unsurprising because, despite conceptual differences, the two sets share many items. Nonetheless, ACR/EULAR criteria were slightly more sensitive, allowing some patients with systemic disease but mild or no sicca symptoms to be classified as having pSS. SGUS was positive in a notable proportion of the patients who received a physician diagnosis of pSS but did not fulfil either criteria set. Thus, including SGUS in the ACR/EULAR criteria may further improve sensitivity.

With the ACR/EULAR criteria, some patients without sicca symptoms may be classified as having pSS if they have systemic features defined by the ESSDAI domains. This point was not specifically addressed during criteria development, because presence of sicca manifestations was required for inclusion in the three different cohorts used to create the criteria [16]. Only three patients in our study met ACR/EULAR criteria despite having no sicca manifestations. These three patients had anti-SSA antibodies and abnormal SGB and received a physician diagnosis of pSS. They had recent-onset disease (with two patients having symptom duration of only 1 year). Although UWSF and Schirmer’s test were normal in these three patients, consistent with the absence of subjective sicca, two patients had abnormal SGUS, suggesting that a pathologic process was developing in their major salivary glands, possibly heralding the subsequent development of sicca manifestations. Prospective longitudinal studies with long follow up will be necessary to assess this hypothesis. Of note, no patient scored positive in the biological ESSDAI domain without having clinical systemic manifestations or sicca symptoms. Among patients who received a physician diagnosis of pSS but did not meet classification criteria, only one had systemic involvement without sicca symptoms. Thus, adding systemic involvement to the criteria, as proposed recently [35], would probably not significantly affect performance of the criteria set in clinical practice [36].

Exclusion of anti-SSB positivity from the ACR/EULAR criteria was based on the finding that anti-SSB-positive/anti-SSA-negative patients in the SICCA cohort lacked key phenotypic features of pSS [37]. In our cohort, only two patients had this serologic profile and both exhibited typical features of pSS and fulfilled ACR/EULAR criteria based on abnormal SGB and objective ocular and oral dryness. In the 2012 ACR classification criteria [11], the combination of positive rheumatoid factor and high-titre anti-nuclear antibodies was proposed as an alternative serologic item for anti-SSA-negative patients but was not selected during the development of the ACR/EULAR criteria. We previously reported that in our cohort, despite an association with pSS diagnosis, this alternative serologic item did not improve classification criteria performance [32].

The AECG criteria include sialography and salivary scintigraphy as objective methods for assessing salivary gland involvement. Neither test was included in the ACR/EULAR criteria. These tests are considered obsolete and are not usually performed in pSS referral centres. Neither test was used in our cohort, and salivary gland dysfunction was defined based only on the UWSF, which is among the ACR/EULAR criteria. However, SGUS is a simple and non-invasive procedure that is readily available to many rheumatologists and supplies important information on the structural changes that develop in the major salivary glands in pSS. Several recent studies found that SGUS exhibited good metrologic properties [28]. In particular, many patients with recent disease already show typical SGUS features, which usually remain stable over the first few years following the diagnosis [38]. Furthermore, SGUS may also be useful as a follow-up tool, as it may help to predict the response to therapy [39] and to detect improvements after active treatment [40]. An international panel of experts was recently established to measure the reproducibility of SGUS and to formally assess the appropriateness of including SGUS among future classification criteria for pSS [41, 42]. Our present analysis suggests that, in addition to UWSF, Schirmer’s test, and the VB score, SGUS may deserve consideration as an alternative objective test for assessing exocrine gland involvement, thereby further increasing sensitivity. Despite lower sensitivity compared to SGB, SGUS brings independent diagnostic data: as we and others previously concluded [23, 43–45], SGUS is not supposed to replace SGB, but could be used as a first step before SGB in the diagnostic algorithm for pSS, and the biopsy could be avoided in anti-SSA+ patients with a positive SGUS.

A recent study from Japan compared the ACR/EULAR criteria, AECG criteria, and Japanese criteria in a multi-centre retrospective cohort of 499 patients with suspected pSS [46]. Agreement was poor. With the physician diagnosis as the reference standard, ACR/EULAR criteria were more sensitive than AECG criteria (95.4% versus 89.4%, respectively) but considerably less specific (72.1% versus 84.3%, respectively). While these sensitivity rates are consistent with ours (87.4% and 82.2% for ACR/EULAR and AECG, respectively), both criteria sets had far lower specificity in the Japanese study than in ours (72.1% versus 95.4% and 89.4% versus 98.1% for ACR/EULAR and AECG, respectively). These findings may indicate important differences in the way physicians diagnose pSS in clinical practice in Japan and in Europe, with Japanese physicians generally identifying pSS cases in clinical practice using Japanese criteria for this disease, which were originally developed as a diagnostic tool [47]. Furthermore, all doubtful cases in our study were reviewed by a panel of three experts to reach a consensus, whereas in the Japanese study [46] the diagnoses were made by the physicians in charge (from ten different hospitals), leaving room for greater variability in the reference standard used to define pSS. Another important point is that stimulated salivary flow (measured by the Saxon test or the gum test) was substituted for UWSF in some patients in the Japanese [46] study, although their diagnostic value is lower than that of UWSF [21].

A limitation of our study is that ocular surface staining (VB score) was performed in only 169 patients (58%). This fact reflects the use of the different diagnostic tests in everyday clinical practice at our centre. However, the VB score was ≥ 4 in only 22.5% of the patients who had this test, including 12 (14.0%) of the 85 patients who met neither criteria set. The vast majority of patients meeting neither criteria set had negative SGB findings and no anti-SSA antibodies and, therefore, would not have fulfilled the criteria even if they had an abnormal VB score. Among the 17 patients who received a physician diagnosis of pSS but fulfilled neither criteria set, only 4 would have fulfilled the ACR/EULAR criteria if they had had a VB available and if this had been positive. It is therefore unlikely that this limitation substantially affected our results.

## Conclusions

In conclusion, in a large cohort of patients with suspected pSS, agreement between the newly developed ACR/EULAR criteria and the earlier AECG criteria was excellent. However, ACR/EULAR criteria were slightly more sensitive and allowed some patients with early disease and prominent systemic features to be classified as having pSS. Our findings also confirm the good metrologic properties of SGUS, suggesting that adding SGUS to classification criteria, as discussed in the report describing the ACR/EULAR criteria [15, 16], may improve classification performance.

## Abbreviations

ACR: American College of Rheumatology
AECG: American-European Consensus Group
Anti-SSA: Anti-Sjögren's-syndrome-related antigen A
ESSDAI: European League Against Rheumatism Sjögren’s Syndrome Disease Activity Index
EULAR: European League Against Rheumatism
OSS: Ocular Staining Score
pSS: Primary Sjögren’s syndrome
SGB: Salivary gland biopsy
SGUS: Salivary-gland ultrasonography
SICCA: Sjögren’s International Collaborative Clinical Alliance
UWSF: Unstimulated whole salivary flow
VB: van Bijsterveld
